# *RGA1* regulates grain size, rice quality and seed germination in the small and round grain mutant *srg5*

**DOI:** 10.1186/s12870-024-04864-5

**Published:** 2024-03-04

**Authors:** Xia Yang, Jun Lu, Wu-Jian Shi, Yu-Hao Chen, Jia-Wen Yu, Sai-Hua Chen, Dong-Sheng Zhao, Li-Chun Huang, Xiao-Lei Fan, Chang-Quan Zhang, Lin Zhang, Qiao-Quan Liu, Qian-Feng Li

**Affiliations:** 1 Jiangsu Key Laboratory of Crop Genomics and Molecular Breeding / Zhongshan Biological Breeding Laboratory/ Key Laboratory of Plant Functional Genomics of the Ministry of Education, College of Agriculture, Yangzhou University, Yangzhou, 225009 Jiangsu China; 2 Jiangsu Key Laboratory of Crop Genetics and Physiology, Jiangsu Co-Innovation Center for Modern Production Technology of Grain Crops, Yangzhou University, Yangzhou, 225009 Jiangsu China

**Keywords:** Rice (*Oryza Sativa*), Grain size, RGA1, Seed germination, Rice quality

## Abstract

**Background:**

Generating elite rice varieties with high yield and superior quality is the main goal of rice breeding programs. Key agronomic traits, including grain size and seed germination characteristics, affect the final yield and quality of rice. The *RGA1* gene, which encodes the α-subunit of rice G-protein, plays an important role in regulating rice architecture, seed size and abiotic stress responses. However, whether *RGA1* is involved in the regulation of rice quality and seed germination traits is still unclear.

**Results:**

In this study, a rice mutant *small and round grain 5* (*srg5*), was identified in an EMS-induced rice mutant library. Systematic analysis of its major agronomic traits revealed that the *srg5* mutant exhibited a semi-dwarf plant height with small and round grain and reduced panicle length. Analysis of the physicochemical properties of rice showed that the difference in rice eating and cooking quality (ECQ) between the *srg5* mutant and its wild-type control was small, but the appearance quality was significantly improved. Interestingly, a significant suppression of rice seed germination and shoot growth was observed in the *srg5* mutant, which was mainly related to the regulation of ABA metabolism. *RGA1* was identified as the candidate gene for the *srg5* mutant by BSA analysis. A SNP at the splice site of the first intron disrupted the normal splicing of the *RGA1* transcript precursor, resulting in a premature stop codon. Additional linkage analysis confirmed that the target gene causing the *srg5* mutant phenotype was *RGA1*. Finally, the introduction of the *RGA1* mutant allele into two indica rice varieties also resulted in small and round rice grains with less chalkiness.

**Conclusions:**

These results indicate that *RGA1* is not only involved in the control of rice architecture and grain size, but also in the regulation of rice quality and seed germination. This study sheds new light on the biological functions of *RGA1*, thereby providing valuable information for future systematic analysis of the G-protein pathway and its potential application in rice breeding programs.

**Supplementary Information:**

The online version contains supplementary material available at 10.1186/s12870-024-04864-5.

## Background

Rice (Oryza sativa L.) is the staple food for more than half of the world’s population. The primary objective of crop breeding is to produce elite rice varieties with high yield and superior quality [1]. The three determinants of rice yield include number of panicles per plant, number of grains per panicle, and 1000-grain weight. Notably, there is a significant positive correlation exists between the grain weight and rice grain size [2, 3]. Grain size is not only a stable agronomic trait, but also plays a crucial role in determining both rice yield and grain quality. At present, more than 400 quantitative trait loci (QTLs) for grain size have been mapped on all 12 chromosomes of rice, and more than 80 grain size-related genes have been successfully cloned [4, 5]. The molecular pathways involved in the regulation of rice grain size can be classified into the following categories, G-protein, ubiquitin-proteasome system, mitogen-activated protein kinase (MAPK) signaling, phytohormones, and transcriptional regulators [6]. In general, grain size genes associated with the ubiquitin-proteasome system, G-protein, and MAPK signaling pathway primarily regulate rice grain size by modulating cell proliferation. However, plant hormones and other signaling pathways influence both cell proliferation and cell expansion to regulate grain size.

Seed dormancy and germination, two distinct physiological processes in seed-bearing plants, are critical to both plant development and crop production. Successful seed germination is an important start to the next round of the plant life cycle, leading to robust and healthy seedling development and ultimately high crop yield. Seed germination begins with the absorption of water and ends with the emergence of the radicle. Appropriate temperatures and adequate water and oxygen availability are required for normal germination [7]. As a key enzyme that catalyzes starch degradation in rice seeds, α-amylase is closely related to several important agronomic traits, such as germination rate [8], seedling vigor [9], and hypoxia stress [10]. Regulation of α-amylase gene expression is a common strategy for coordinated plant growth and development. In barley, abscisic acid (ABA) antagonizes gibberellin (GA) by inhibiting GAMYB expression and subsequent a-amylase expression, thereby modulating seed dormancy and germination [11]. Brassinosteroids (BRs), a major plant growth-promoting hormone, are also involved in the regulation of seed germination [12, 13]. In Arabidopsis, BR promoted seed germination by antagonizing the inhibitory effect of ABA, mechanistically by targeting ABSCISIC ACID-INSENSITIVE5 (ABI5) [14, 15].

As an important regulator of grain size, the G protein is composed of heterotrimers of Gα, Gβ, and Gγ subunits, and acts as a signaling coupler between the receptor in the membrane and the effector in the cytoplasm through the exchange of GDP for GTP on the Gα subunit [16]. The plant Gα subunit has been successfully cloned in both Arabidopsis and rice [17, 18]. The rice G-α subunit is encoded by the *RGA1* gene, whose promoter contains a putative TATA box, a potential CAAT box, and an ABRE cis-acting element, known to be involved in ABA-induced expression [19]. In addition, the *RGA1* genomic sequence contains thirteen exons and twelve introns, and a point mutation (G to A) has been identified at the splicing site (GT-AG) of the first intron, resulting in the generation of abnormal mRNA splicing forms [20]. In rice, the *RGA1* gene plays important roles in a number of plant developmental events and stress responses [18, 21–23], including plant height [23], hypoxia stress [24], salt-induced cell senescence and division [16], cold and heat stress [25], and drought stress [26]. In addition, RGA1 can also enhance rice yield under abiotic stress by modulating photoprotection and photoavoidance through the G-protein pathway [23]. The increasing number of studies focusing on the investigation of *RGA1* gene in rice suggests the important role of RGA1 in the regulation of rice growth and development, especially its involvement in abiotic stress responses. However, there are still no reports on whether RGA1 is involved in the regulation of rice quality, seed dormancy, and germination traits.

In this study, a rice mutant *srg5* (small and round grain 5) with reduced plant height and small grain was identified in an EMS-induced rice mutant library. First, its major agronomic traits determining rice yield, grain quality, and germination characteristics were systematically analyzed. Then, BSA assay and linkage analysis were performed and successfully identified *RGA1* as the target gene of the *srg5* mutant. Finally, the introduction of the *RGA1* mutant allele into two indica rice varieties with slender grain shape all resulted in round rice grains, i.e., reduced grain length and increased grain width. Thus, this study sheds new light on the biological functions of *RGA1* and provides valuable information for our understanding of the G-protein pathway and for breeding elite rice varieties using the *RGA1* gene.

## Results

### *srg5* mutant with round grain shape

**Fig. 1:**
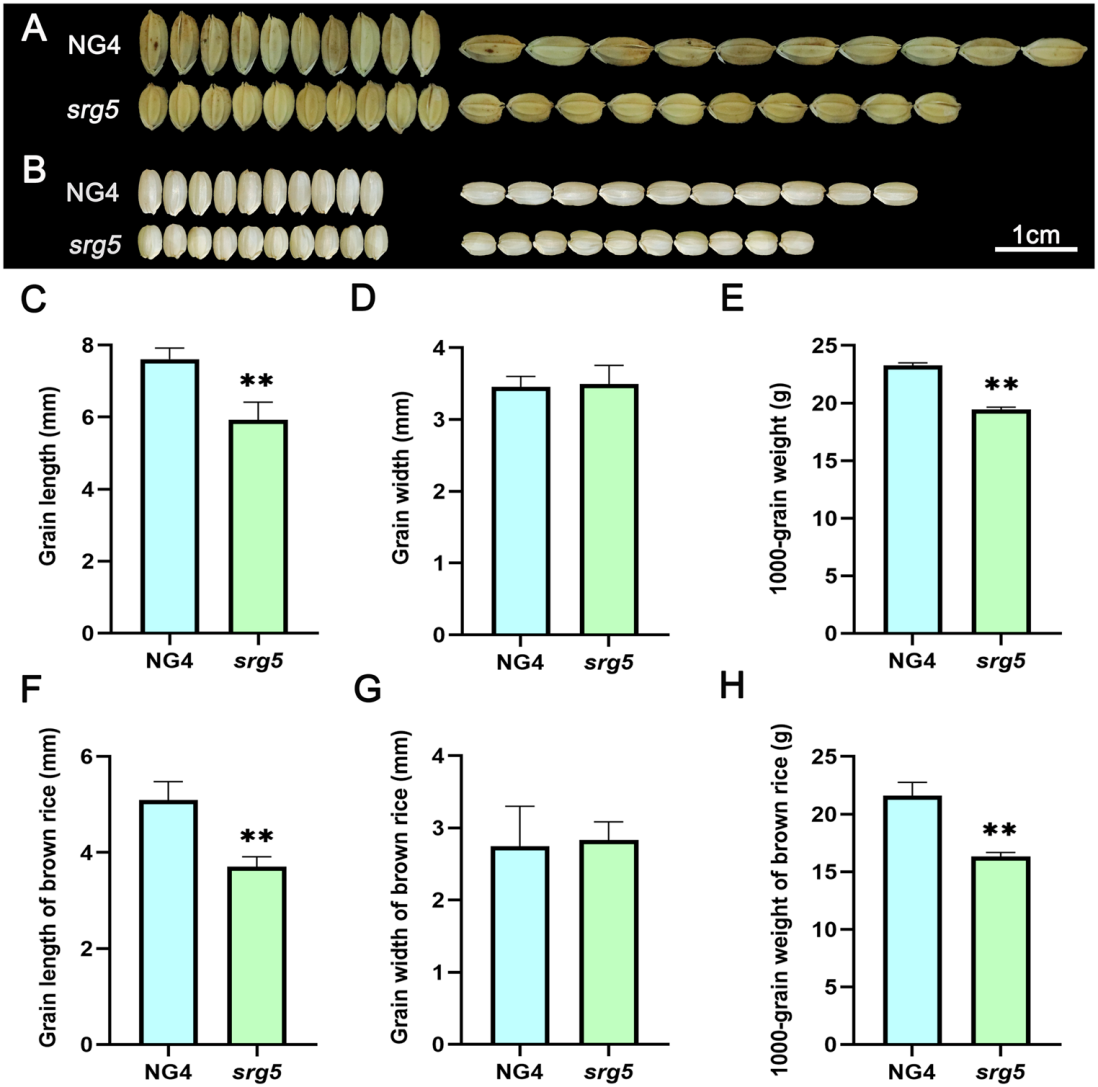
Grain size analysis of *srg5* mutant and its wild type NG4. Grain length and width (**A**), brown rice length and width (**B**) of *srg5* mutant and the NG4 control. Quantitative data for grain length (**C**), grain width (**D**) and 1000-grain weight (**E**) of *srg5* mutant and the NG4 control. Quantitative data for length (**F**), width (**G**) and 1000-grain weight (**H**) of the brown rice of *srg5* mutant and the NG4 control. Error bars represent the SDs. **P < 0.01 (Student’s t-test)

Compared with wild-type NG4, the *srg5* mutant exhibited significantly reduced grain length and slightly increased grain width (Fig. 1A, C and D), thus resulting in an extremely small and round rice grain. As expected, the 1000 grain weight of the mutant was also significantly reduced due to the small grains (Fig. 1E). Correspondingly, the *srg5* mutant brown rice showed a similar seed size in terms of grains (Fig. 1B, F-H).

### The *srg5* mutant was semi-dwarf, with a reduction in data for most of the agronomic traits tested

**Fig. 2:**
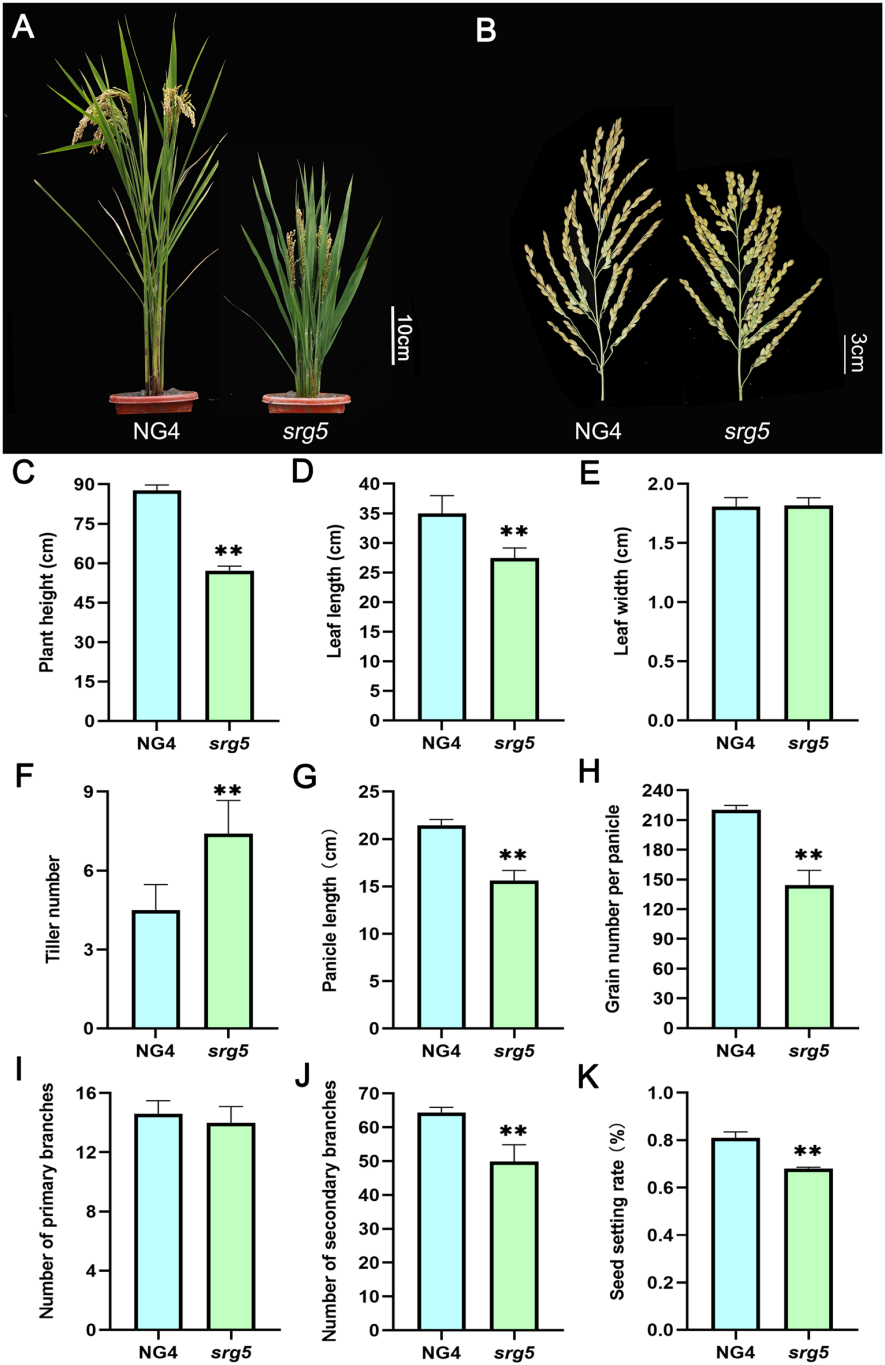
Analysis of the main agronomic traits of the *srg5* mutant and the control NG4. Plant architecture (**A**) and panicle phenotype (**B**) of the *srg5* mutant and NG4. Quantitative data of plant height (**C**), leaf length (**D**), leaf width (**E**), number of tillers (**F**), panicle length (**G**), number of grains per panicle (**H**), number of primary branches (**I**), number of secondary branches (**J**), and seed setting rate (**K**) of the *srg5* mutant and the control NG4. Error bars represent the SDs. ^**^P < 0.01 (Student’s t-test)

The main agronomic traits of the *srg5* mutant and the wild-type control were systematically analyzed at the maturity stage. In general, the *srg5* mutant showed a semi-dwarf phenotype with significant reduction in plant height, leaf length, and panicle length (Fig. 2A-D, G). However, its leaf width was unchanged and the number of tillers was even increased (Fig. 2E, F). In addition, the number of grains per panicle was reduced in the *srg5* mutant, mainly due to the reduced number of secondary branches instead of primary branches (Fig. 2H-J). The seed setting rate of the *srg5* mutant was also lower than that of the wild-type control (Fig. 2K). All these changes in seed and panicle traits resulted in a remarkable reduction in grain yield per plant in the *srg5* mutant (Fig. S1).

### Chalkiness of *srg5* seeds was reduced

**Fig. 3:**
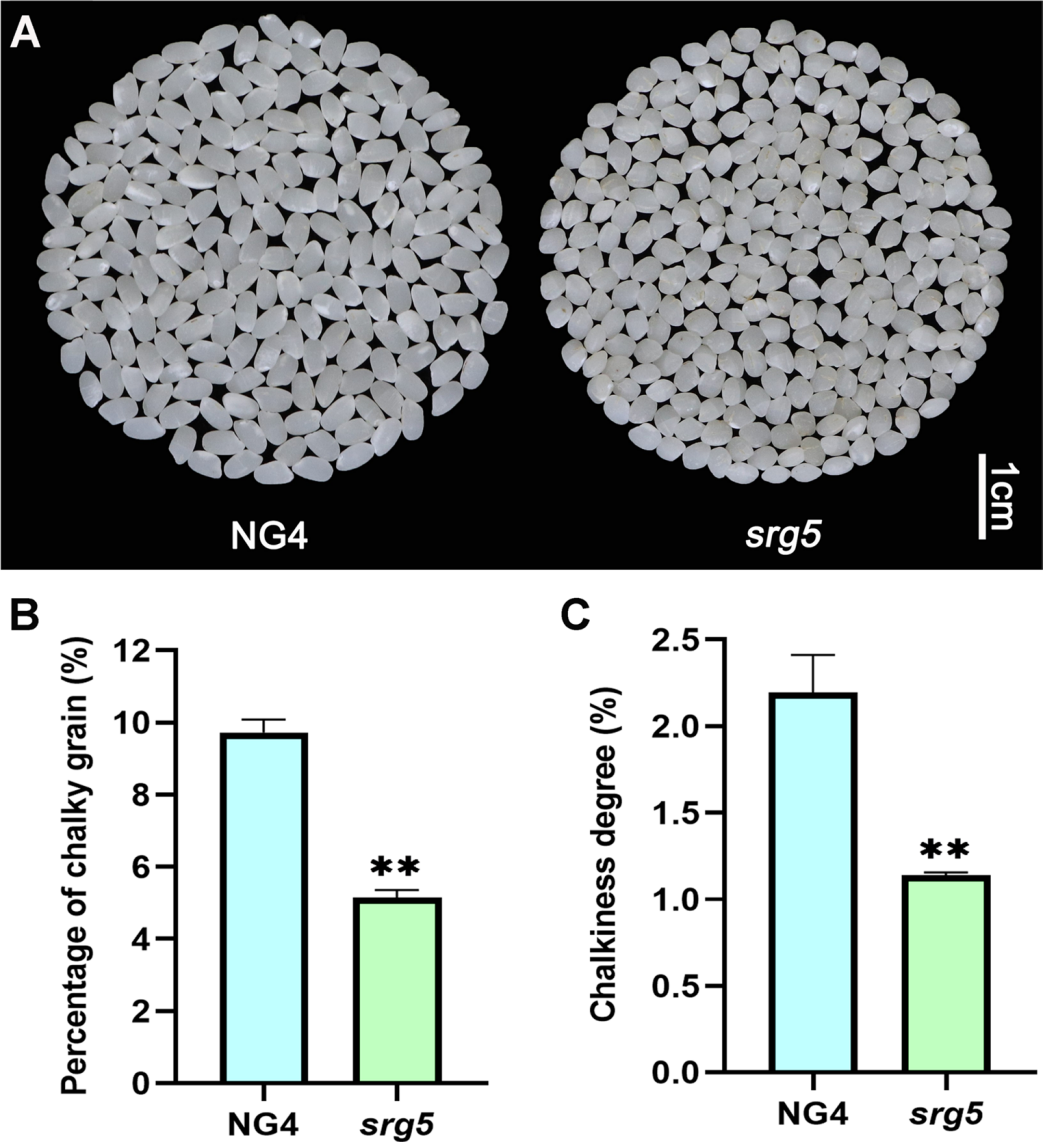
Analysis of the appearance quality of the *srg5* mutant and the wild-type control. (**A**) Comparison of the appearance of milled rice between the *srg5* mutant and NG4. Percentage of chalky grains (**B**) and chalkiness degree (**C**) of the *srg5* mutant and NG4. Error bars represent the SDs. ^**^P < 0.01 (Student’s t-test)

Grain size not only affects rice yield but also influences rice appearance quality. In general, rice grains with a slender shape will have less chalkiness [27]. Interestingly, although the *srg5* mutant showed a round grain shape with significantly reduced grain length and even slightly increased grain width (Fig. 1A-D, F and G), both the percentage of chalky grains and the chalkiness degree of the *srg5* mutant were significantly reduced (Fig. 3A-C), suggesting that the appearance quality of the *srg5* mutant was improved.

### Physicochemical properties of the *srg5* seeds were slightly modified

**Fig. 4:**
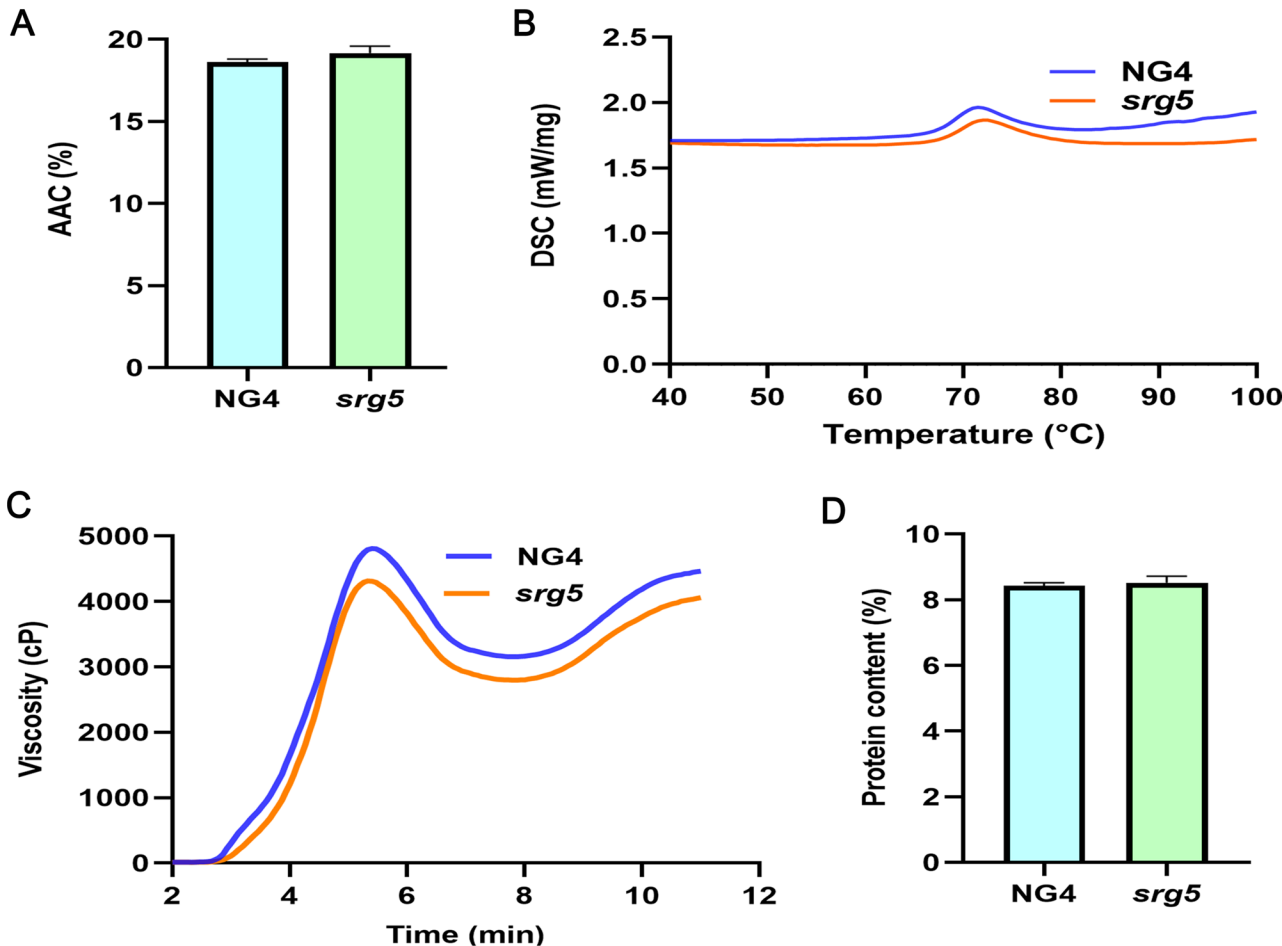
Physicochemical analysis of the *srg5* mutant and the NG4 control. (**A**) Apparent amylose content (AAC), (**B**) gelatinization characteristics, (**C**) pasting characteristics, and (**D**) protein content of *srg5* mutant rice and the wild-type control. Error bars represent the SDs

To systematically evaluate the eating and cooking quality of the *srg5* mutant, the main physicochemical properties of rice seeds were investigated. In general, the physicochemical properties tested were unchanged or slightly modified in the *srg5* mutant. In particular, the apparent amylose content (AAC) and protein content were similar between the *srg5* mutant and the NG4 control (Fig. 4A, D). Regarding thermal properties, DSC analysis showed that the onset temperature (To) and peak temperature (Tp) of the *srg5* mutant were reduced (Fig. 4B; Table S1). Viscosity can simulate dynamic changes during rice cooking process and reflect rice texture. The RVA assay showed that the data of all parameters had some decrease in the *srg5* mutant, but only the hot paste viscosity (HPV) was significantly decreased (Fig. 4C; Table S2). All these data indicated that the *SRG5* mutation slightly affected the thermal properties and viscosity properties of rice.

### Germination of *srg5* seeds was delayed

**Fig. 5:**
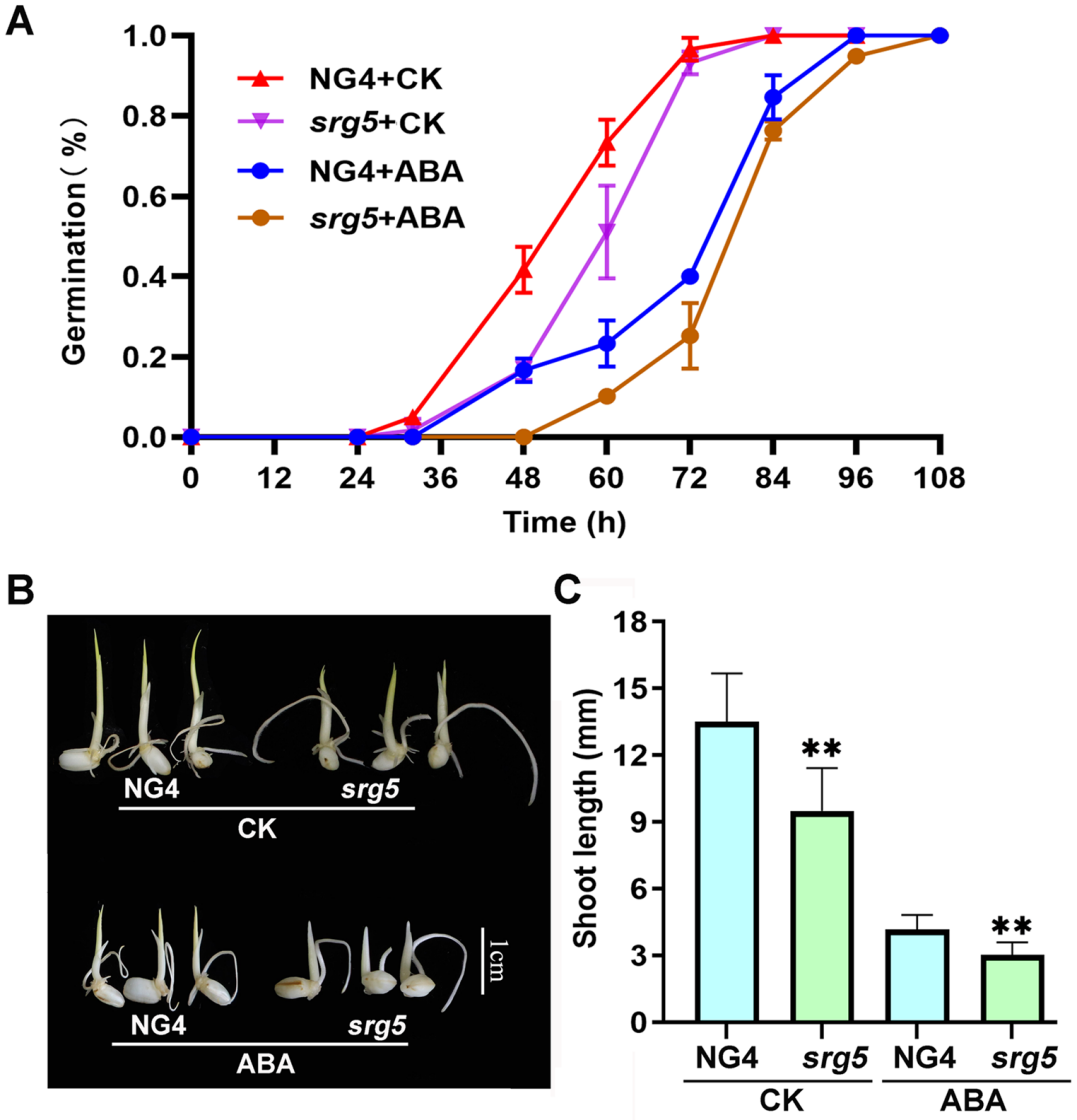
Seed germination analysis of the *srg5* mutant and the NG4 control. (**A**) Seed germination analysis with ABA or mock treatment. (**B**) Shoot length of germinating seeds with or without ABA treatment. (**C**) Quantitative data of the shoot length of the germinating seeds. The concentration of ABA for treatment was 1 µM. The shoot lengths of the germinating seeds were measured 96 h after imbibition (HAI). Error bars represent the SDs. **P < 0.01 (Student’s t-test)

To investigate whether the *SRG5* mutation affects rice seed germination, we performed seed germination analysis. Compared with wild-type NG4, the *srg5* mutant showed a slower germination rate, but the final germination rate was not affected. ABA treatment delayed the germination process of both the *srg5* mutant and the NG4 control (Fig. 5A), indicating that the *SRG5* mutation didn’t affect the sensitivity of rice to ABA. In addition, the *srg5* mutant showed a significantly shorter bud length compared to the wild-type control under both normal and ABA-treated germination conditions (Fig. 5B, C). Therefore, the *SRG5* mutation inhibits rice germination rate and shoot growth.

### ABA levels in *srg5* mutant seeds were significantly increased

**Fig. 6:**
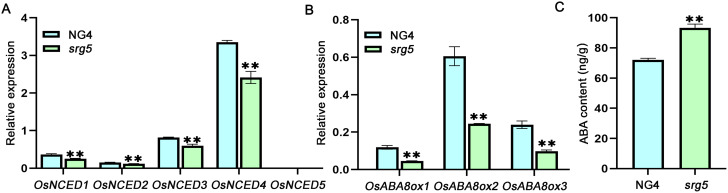
Analysis of ABA accumulation in the *srg5* mutant. Expression analysis of ABA biosynthesis genes (**A**) and metabolic genes (**B**), and quantification of ABA levels (**C**) in the *srg5* mutant and the NG4 control. Error bars represent the SDs. **P < 0.01 (Student’s t-test)

To explore the possible mechanism of the delayed seed germination of the *srg5* mutant, the expression of ABA metabolism genes was analyzed. Interestingly, RT-qPCR data indicated that the expression of all tested key genes involved in ABA biosynthesis and metabolism was decreased in the *srg5* mutant (Fig. 6A, B). Specifically, the expression of most of the ABA biosynthesis genes in the mutant was about 70% of that in the wild-type control, while the expression of all three ABA metabolism genes in the mutant was only about 40% of that in the NG4 control (Fig. 6A, B), suggesting that the *SRG5* mutation resulted in a more pronounced suppression effect on the ABA metabolism pathway. To validate this hypothesis, we directly measured the ABA levels in the germinating seeds of the *srg5* mutant and the wild-type control. The result showed that there was an approximately 20% increase in ABA levels in the *srg5* mutant compared to the wild type control. Therefore, the delayed germination of the *srg5* mutant was, at least in part, due to the increased ABA content in the seeds resulting from the decreased expression of ABA metabolic genes (Fig. 6C).

### Identification of *SRG5* target gene

**Fig. 7:**
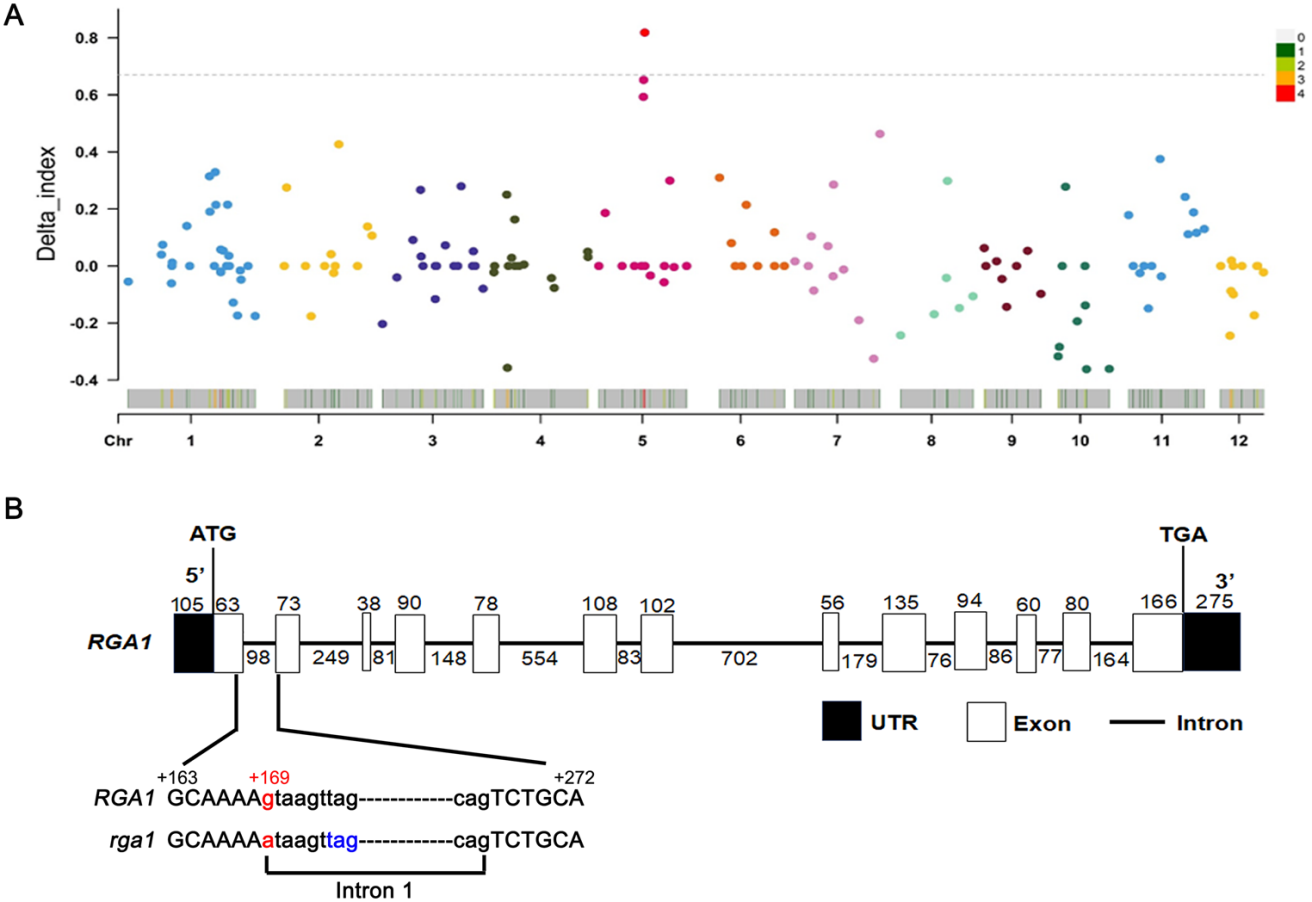
BSA analysis and the gene structure of *SRG5/RGA1*. (**A**) BSA analysis. The x coordinate is the name of the chromosome in the rice genome, and the dashed line in the middle represents the threshold value of significant association. The higher the delta index value, the better the association effect at that point in the table. (**B**) Diagram of the *SRG5/RGA1* gene structure and the mutation site of *RGA1*. The SNP is shown in red and the premature stop codon in the *rga1* allele is shown in blue

To identify the target gene of *SRG5*, BSA analysis was performed. The *srg5* mutant was backcrossed with the wild-type control and the resulting F_2_ population was used to select plants with small grain or normal grain, respectively, forming the two populations for the BSA assay (Fig. 7A). Candidate gene screening was performed as follows. First, the SNP index value of the SNP site approached the significance threshold of 0.68. Second, the identified SNP should be induced by EMS mutagenesis, specifically from G to A or C to T. Third, if the SNP was located in the coding region, it would cause premature termination or at least an amino acid substitution. BSA analysis showed that two candidate SNPs met these criteria. Both SNPs were located on chromosome 5 and corresponded to two candidate genes, Os05g0333200 and Os05g2617000, respectively (Table S3). Based on the gene annotations, Os05g0333200 encodes a G protein α-subunit RGA1, and Os05g2617000 encodes a retrotransposon protein.

To further identify the target gene responsible for the small-grain phenotype of the *srg5* mutant, genetic linkage analysis was performed on a subset of 20 normal-grain and 20 small-grain plants from the F_2_ population. The results showed that in plants with normal grain size, the *RGA1* gene had either a homozygous G/G genotype or a heterozygous G/A genotype, whereas all small-grain plants had a homozygous A/A genotype. For the gene encoding the retrotransposon protein, the homozygous G/G genotype and the heterozygous G/A genotype were present in the normal grain plants. However, both homozygous A/A genotype and heterozygous G/A genotype existed in small grain plants. Therefore, the *RGA1* gene should be the target gene of the *srg5* mutant (Fig. 7B) [28]. Further sequence analysis revealed that the identified SNP was located in the first intron of *RGA1*, which was consistent with the previous publication. Specifically, a G to A mutation occurred at the GT-AG splice site of the first intron, disrupting the normal splicing of the *RGA1* transcript precursor and resulting in a premature stop codon (Fig. 7B). The resulting mutant protein contained only 23 amino acids, whereas the wild-type RGA1 protein contained 380 amino acids (Fig. S2). The SNP ultimately led to the phenotypic changes, such as small grain size and reduced plant height. Therefore, *RGA1* is considered to be the target gene of the *srg5* mutant.

### Introduction of the *rga1* allele into indica rice also resulted in small and round grains

**Fig. 8:**
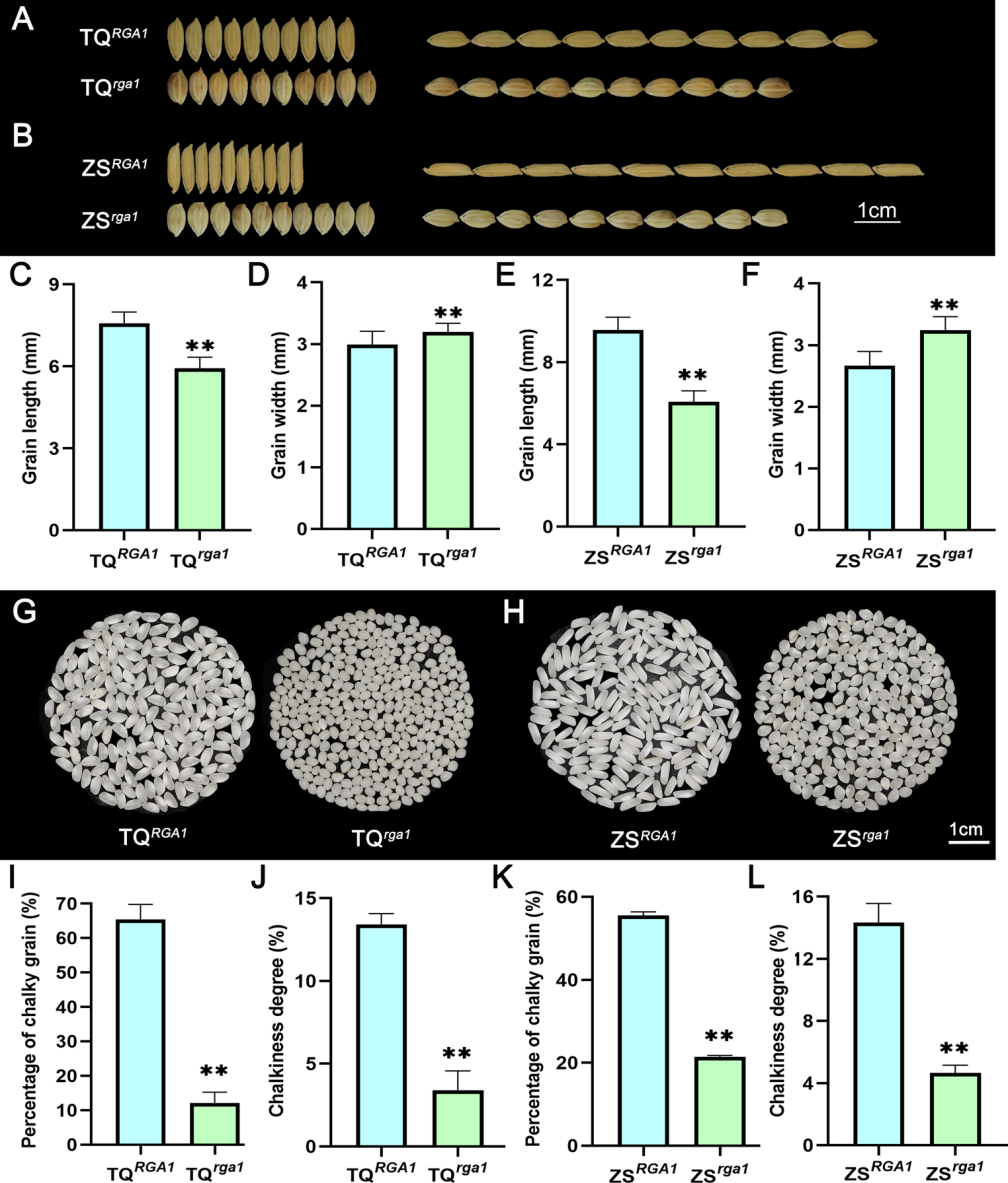
Grain size analysis of indica rice varieties with *rga1* allele. (**A**) Grain shape of indica rice Teqing (TQ) and TQ^*rga1*^. (**B**) Grain shape of indica rice Zhenshan 97B (ZS) and ZS^*rga1*^. Quantitative data of the grain length (**C**) and grain width (**D**) of TQ and TQ^*rga1*^. Quantitative data of the grain length (**E**) and grain width (**F**) of ZS and ZS^*rga1*^. (**G**) Comparison of the appearance of milled rice between TQ and TQ^*rga1*^. (**H**) Comparison of the appearance of milled rice between ZS and ZS^*rga1*^. Percentage of chalky grains (**I**) and chalkiness degree (**J**) of TQ and TQ^*rga1*^. Percentage of chalky grains (**K**) and chalkiness degree (**L**) of ZS and ZS^*rga1*^. Error bars represent the SDs. **P < 0.01 (Student’s t-test)

To further evaluate the effect of the *rga1* allele on grain size in indica rice, we crossed the *srg5* mutant with two indica rice varieties, namely Teqing (TQ), and Zhenshan 97B (ZS). We then carefully selected several plants from the F_2_ segregation population with similar small grain shape and plant height similar to the *srg5* mutant. These selected rice plants were then self-crossed for 3 to 4 consecutive generations. Three to four recombinant inbred lines with stable phenotypes were then successfully selected for each hybrid combination. The *RGA1* loci of these small-grain recombinant inbred lines were then sequenced, confirming the presence of the same type of mutation as observed in the *srg5* mutant. The result of grain size analysis showed that all grains of the selected recombinant inbred lines exhibited small and round grain shape, with significantly reduced grain length and increased grain width compared to those of their indica parents (Fig. 8A-F). Correspondingly, the 1000-grain weight of both TQ^*rga1*^ and ZS^*rga1*^ was significantly reduced compared to the corresponding wild-type control (Fig. S3). In addition, the percentage of chalky grains and the chalkiness degree of the TQ^*rga1*^ and ZS^*rga1*^ rice were also significantly reduced (Fig. 8G-L), suggesting that the introduction of the *rga1* allele can improve the appearance quality of indica rice. Taken together, all these data further support the notion that *RGA1* is indeed the target gene of the *srg5* mutant.

## Discussion

Rice yield is a multifaceted quantitative trait controlled by a large number of factors and genes [29]. In recent years, significant progress has been made in identifying key genes involved in the regulation of grain number and weight, which has greatly facilitated the genetic improvement of rice yield. For example, OsMADS17 can directly interact with the promoter of *OsAP2-39* and regulate its expression. On the other hand, the expression of *OsMADS17* itself is directly regulated by the transcription factor OsMADS1, thus revealing an OsMADS1-OsMADS17-OsAP2-39 molecular module in the control of rice yield [30]. Grain size affects not only rice yield but also the quality of rice appearance [31, 32]. To date, more than 80 rice grain size genes have been cloned and mechanistically implicated in several regulatory pathways. One important pathway is the G-protein signaling pathway, including *GS3* [33–35], *DEP1/qPE9* [36, 37], *RGG1* [38], *RGG2* [39], *GGC2* [40] and *RGA1* [22, 34]. The *RGA1* gene, which encodes the alpha subunit of the G protein, is involved in the regulation of a number of seed-related traits in rice, such as grain size, rice eating and cooking quality, rice chalkiness, and seed germination characteristics (Figs. 1, 3, 4, 5 and 8). Impressively, we have shown that RGA1 positively regulates seed germination mainly by modulating ABA content (Fig. 6).

In addition to its role in controlling seed traits in rice [41], RGA1 has been reported to mediate rice responses to abiotic stress and plant hormones, including cold and heat stress, salt stress, drought stress, plant immunity, photooxidation, and BR signaling [42–44]. For example, the whole transcriptome microarray analysis was performed using the *RGA1* null mutant *d1* and its wild type Nipponbare. A total of 2270 unique differentially expressed genes (DEGs) were identified, including 111 heat stress genes, 8 cold stress genes, 8 drought stress genes and 2 salt stress genes, suggesting that *RGA1* may play a broad role in the regulation of various abiotic stresses in rice [45]. Under salt stress, *RGA1* enhances the salt tolerance of the *d1* mutant by modulating the expression of genes involved in metabolic pathways, photosynthesis, and reactive oxygen species (ROS) homeostasis, resulting in the reduction of ROS accumulation [20]. In addition, net carbon fixation of wild-type rice ceased 11 days after water deprivation, whereas the *d1* mutant exhibited sustained net photosynthesis for an additional week. Furthermore, under drought stress conditions, the reduced leaf temperature in the *d1* mutant resulted in a lower driving force for water loss. Therefore, as drought stress intensified, the rate of decline of photosynthesis in the *d1* mutant was slowed, thereby enhancing its drought tolerance [26]. In addition, RGA1 also functions as a key regulator of photoinhibition and photoprotection in rice, thereby alleviating persistent photoinhibition damage and enhancing the abiotic stress tolerance in rice [23]. A recent report showed that RGA1 can improve energy metabolism and distribution in rice by facilitating sucrose unloading in the pistils, thereby mitigating the detrimental effects of low light stress on pollen tube elongation and enhancing rice panicle fertility [46]. Recently, a nitrate-responsive transcriptome assay was performed and showed that RGA1 plays a negative role in regulating nitrogen sensitivity and use efficiency in rice [45]. Although a number of studies have revealed the function of RGA1 in various stress responses and plant growth, its role in regulating rice quality and germination traits has not been reported so far. Our study helps to elucidate the role of *RGA1* in controlling various aspects of yield and quality traits in rice.

## Conclusions

In this study, the effects of the *RGA1* mutation on rice grain size, plant morphology, panicle characteristics, eating and cooking quality (ECQ), and seed germination characteristics were comprehensively investigated. The results showed that the *RGA1* mutation resulted in a semi-dwarf rice plant with small grain size and reduced panicle length, which was consistent with the previous reports. In addition, this study showed that the *RGA1* mutation had little effect on rice ECQ, but significantly reduced rice germination speed and shoot growth by modulating ABA metabolism. Furthermore, we demonstrated that the mutant *rga1* allele also functions in the control of grain size in the genetic background of indica rice, and its regulatory effect depends on the length-width ratio of the original rice seed. Taken together, this study further enhances our understanding of the function and effect of *RGA1* in the regulation of rice growth and development, thereby providing valuable information for future systematic analysis of the G-protein signaling pathway and its potential application in rice breeding.

## Materials and methods

### Rice materials

A japonica rice variety, Ninggeng 4 (NG4), was treated with ethylene methyl sulfonate (EMS) and an *srg5* mutant with short plant height and small grain size was identified. Several generations of self-pollination showed that the phenotype of the *srg5* mutant was stable. All rice materials were grown in the paddy field of Yangzhou University (Yangzhou, Jiangsu Province, China) in summer. The standard wide-narrow row planting method was used for all rice materials under conventional crop management.

### Analysis of main agronomic characteristics of rice

The main agronomic characteristics of rice, including plant height, leaf length and width, tiller number, panicle length, grain number per panicle, seed setting rate, 1000-grain weight, were measured and recorded after seed maturity. Mature rice seeds from superior rice panicles were collected and air-dried, and the rice grain length and width were measured using the rice appearance quality detector (SC-E, Wanshen, China).

### Evaluation of rice chalkiness

Air-dried mature rice grains were first dehusked using a rice huller (model SY88-TH, South Korea). Then any moldy or immature grains were manually selected and removed from the brown rice. Next, the dehusked rice were polished using a Kett grain polisher (Tokyo, Japan). Finally, the chalkiness trait of the milled rice seeds was evaluated by using the Wanshen SC-E rice seed detector.

### Rice flour preparation and physicochemical analyses

The polished rice was ground into fine flour using a mill (FOSS 1093 Cyclotec Sample Mill, Denmark) equipped with a 0.15 mm sieve. The rice flour samples were then dried at 40 °C for 3 days, followed by another 3 days of equilibration at room temperature. The treated samples were then sealed and stored at 4 °C for subsequent rice quality analysis [47].

The rice apparent amylose content (AAC) of rice was determined by the iodine colorimetric method [48]. The thermodynamic properties of rice were determined using a differential scanning calorimeter (DSC 200 F3, Netzsch Instruments NA LLC; Burlington, MA) [49]. The viscosity of rice was measured using a Rapid Visco Analyzer (RVA) (Techmaster; Newport Scientific, Australia), and the data obtained were analyzed using the TCE software [50].

### Total protein analysis

The Lowry Protein Assay Kit (PC0030, Solarbio) was used to determine the total soluble protein content of rice. Briefly, 20 mg of processed rice flour was accurately weighed and transferred to a 2 ml centrifuge tube. Then 0.1 ml of ethanol was added, followed by 1.8 ml of 1 M NaOH solution. After mixing, the samples were placed vertically in a shaker at 200 rpm for 3 h (50 °C). Then 100 µl dispersion was added to 300 µl PBS buffer. At the same time, an appropriate amount of BSA protein was used to construct a standard curve. 20 µl of the diluted dispersion was transferred to an ELISA plate and 200 µl of prepared Folin Phenol A reagent was added to the sample well. After gentle mixing, the sample was incubated at 37 °C for 10 min. Then 20 µl of Folin Phenol B reagent was added to each well and incubated at 37 °C for 30 min. The absorbance of the samples was measured at the wavelength of 750 nm, and finally a standard curve was constructed to calculate the protein content.

### Analysis of seed germination

The mature rice seeds were manually dehulled and used for seed germination analysis. The detailed procedure can be found in the previous publication [51].

### BSA analysis for mapping the target gene of the *srg5* mutant

The *srg5* mutant was backcrossed with the wild-type NG 4, and the resulting F_1_ hybrid was self-pollinated to obtain the F_2_ segregating population. Grain length, grain width, and the length-width ratio of each F_2_ rice plant were evaluated, while segregation ratios of the subsequent generations were analyzed using the continuity-corrected chi-squared test.

By analyzing the grain size of F_2_ plants, 30 plants with extremely small grain size and 30 plants with normal grain size were selected to form two populations for the following BSA assay. An equal amount of genomic DNA from each plant of the same population was mixed into a control DNA pool and a mutant DNA pool. Then, two parents, *srg5* mutant and the wild-type NG4, were re-sequenced at a 10 × sequencing depth, and the two mixed DNA pools were re-sequenced at a 30 × sequencing depth (Novogene Co., Ltd.). The reference rice genome used for the analysis was Japonica rice Nipponbare (https://rapdb.dna.affrc.go.jp/download/irgsp1.html).

Based on the result of BSA analysis, two candidate genes were identified. Then, two more sets of F_2_ rice samples, each set containing 20 plants with small grain size and normal grain size, respectively, were used for linkage analysis and the final determination of the target gene.

### Total RNA isolation and RT-qPCR analysis

Total RNA was extracted from rice samples using the RNA simple Total RNA Kit (Tiangen, Beijing, China). A total of 1 µg RNA was used for reverse transcription by using the HiScript III 1st Strand cDNA Synthesis Kit (+ gDNA wiper) (Vazyme, Nanjing, China). Gene expression analysis was performed on the CFX96 Touch real-time PCR detection system (Bio-Rad, California, USA) using the ChamQ Universal SYBR qPCR Master Mix Kit (Vazyme, Nanjing, China). The *Actin* gene was used as an internal control for gene expression analysis. All the related primers for RT-qPCR assay are listed in Table S4.

### Data processing

Statistical data in this study are presented as mean ± standard deviation (SD). Student’s t-test was used to determine the significance of single pairwise comparisons (* *p* < 0.05; **p < 0.01). Significant differences at the 0.05 level for multiple comparisons are indicated by different lowercase letters.

### Electronic supplementary material

Below is the link to the electronic supplementary material.

Supplementary Material 1

## Data Availability

No datasets were generated or analysed during the current study.
