# Supplementary material for: RGA1 regulates grain size, rice quality and seed germination in the small and round grain mutant srg5
Source: BMC Plant Biol. 2024 Mar 4;24:167. doi: 10.1186/s12870-024-04864-5 (PMC10910726; doi:10.1186/s12870-024-04864-5)
Supplement: Supplementary file 1 — Supplementary Material 1 [file 12870_2024_4864_MOESM1_ESM.pdf]

Supplementary Information

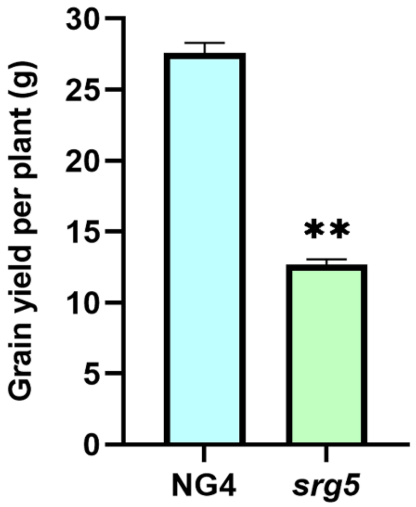

Fig.S1. Grain yield analysis of the *srg5* mutant and its wild-type control.

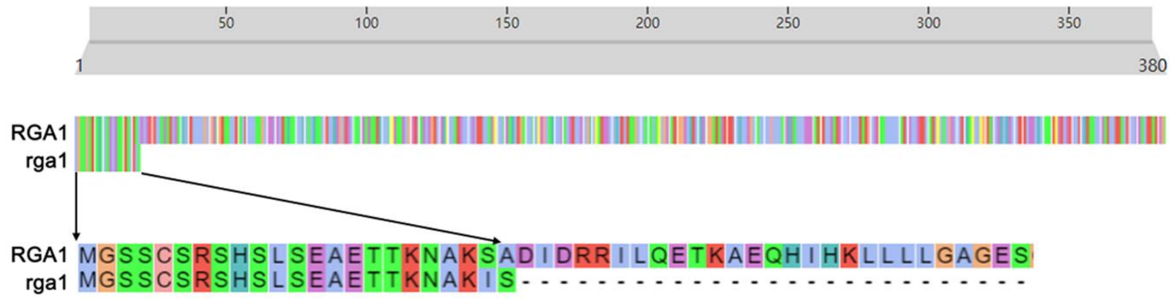

Fig.S2. Amino acid sequence comparison between RGA1 and rga1.

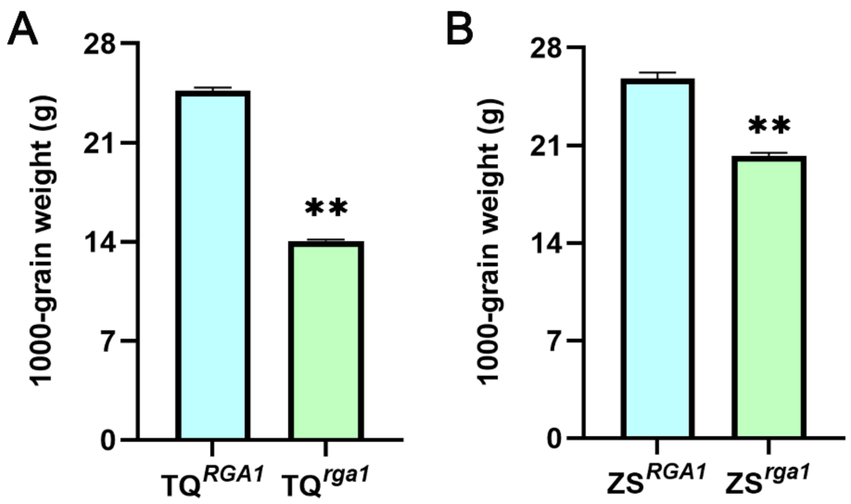

Fig.S3. Grain weight analysis of TQ^{rga1} and ZS^{rga1} and their corresponding wild type.

Table S1. DSC-related parameters of the *srg5* mutant and its wild-type control

Sample name	Enthalpy ΔH (J/g)	Onset temperature To (°C)	Peak temperature Tp (°C)	Conclusion temperature Tc (°C)
NG4	8.45±0.10	66.43±0.35	71.63±0.20	77.40±0.32
srg5	8.31±0.09	65.40±0.00*	70.80±0.00*	76.90±0.70

Data are means ± SD. * P < 0.05 (Student's t-test).

Table S2. RVA-related parameters of the *srg5* mutant and its wild-type control

Sample	Peak viscosity PKV (cP)	Hot paste viscosity HPV (cP)	Breakdown BDV (cP)	Cool paste viscosity CPV (cP)	Setback value SBV (cP)
NG4	4633.50±36.06	2937.50±26.16	1696±9.89	4359±46.66	-274.5±17.67
srg5	4535.5±28.99	2772.5±37.47*	1569.5±28.99	4174±31.11	-319±8.48

Data are means ± SD. * P < 0.05 (Student's t-test).

Table S3. *SRG5* candidate genes identified by BSA analysis

Chr	WT	Mutant	Delta-index	Gene ID	Mutation site	Annotation
5	C	T	0.82	Os05g0333200	Intron 1	*d1*，*RGA1*
5	C	T	0.65	Os05g2617000	3`UTR	retrotransposon protein

Table S4. Primers used in this study

Primer Name	Primer sequence
OsNCED1-F	TCGCCATCACCGAGAACTA
OsNCED1-R	TCTCCTGGAGCTTGAACACC
OsNCED2-F	GGTATGGAAACGAGGATAGTGGTT
OsNCED2-R	TGCTTATTGTTGTGCGAGAAGTTC
OsNCED3-F	CCCCTCCCAAACCATCCAAACCGA
OsNCED3-R	TGTGAGCATATCCTGGCGTCGTGA
OsNCED4-F	TCCATCTCCTTCTCCCTCCTCCCA
OsNCED4-R	CCTCGCACCCTGCTTGATCTTGCC
OsNCED5-F	ACATCCGAGCTCCTCGTCGTGAA
OsNCED5-R	TTGGAAGGTGTTTTGGAATGAACCA
OsABA80X1-F	AAGCTGGCAAAACCAACATC
OsABA80X1-R	TGGATTCCGTATTAGCACGG
OsABA80X2-F	GCGAGACGCTCCAGCTCT
OsABA80X2-R	GGGCACCCCAGCAGATT
OsABA80X3-F	AGTACAGCCCATTCCCTGTG
OsABA80X3-R	ACGCCTAATCAAACCATTGC
OsActin-F	CCAAGGCCAATCGTGAGAAGA
OsActin-R	AATCAGTGAGATCACGCCCAG
